# Mapping the daily rhythmic transcriptome in the diabetic retina

**DOI:** 10.1016/j.visres.2023.108339

**Published:** 2023-11-30

**Authors:** Ryan P. Silk, Hanagh R. Winter, Ouria Dkhissi-Benyahya, Carmella Evans-Molina, Alan W. Stitt, Vijay K. Tiwari, David A. Simpson, Eleni Beli

**Affiliations:** a Wellcome Wolfson Institute for Experimental Medicine, Queens’ University Belfast, Northern Ireland, United Kingdom; b Univ. Lyon, Université Lyon 1, Inserm, Stem Cell and Brain Research Institute U1208, 69500 Bron, France; c Center for Diabetes and Metabolic Disease, Indiana University School of Medicine, Indianapolis, IN, USA; d Institute of Molecular Medicine, University of Southern Denmark, Odense C, Denmark; e Danish Institute for Advanced Study (DIAS), Odense M, Denmark; f Department of Clinical Genetics, Odense University Hospital, Odense C, Denmark

## Abstract

Retinal function changes dramatically from day to night, yet clinical diagnosis, treatments, and experimental sampling occur during the day. To begin to address this gap in our understanding of disease pathobiology, this study investigates whether diabetes affects the retina’s daily rhythm of gene expression. Diabetic, Ins2^Akita/J^ mice, and non-diabetic littermates were kept under a 12 h: 12 h light/dark cycle until 4 months of age. mRNA sequencing was conducted in retinas collected every 4 h throughout the 24 hr light/dark cycle. Computational approaches were used to detect rhythmicity, predict acrophase, identify differential rhythmic patterns, analyze phase set enrichment, and predict upstream regulators. The retinal transcriptome exhibited a tightly regulated rhythmic expression with a clear 12-hr transcriptional axis. Day-peaking genes were enriched for DNA repair, RNA splicing, and ribosomal protein synthesis, night-peaking genes for metabolic processes and growth factor signaling. Although the 12-hr transcriptional axis is retained in the diabetic retina, it is phase advanced for some genes. Upstream regulator analysis for the phase-shifted genes identified oxygen-sensing mechanisms and HIF1alpha, but not the circadian clock, which remained in phase with the light/dark cycle. We propose a model in which, early in diabetes, the retina is subjected to an internal desynchrony with the circadian clock and its outputs are still light-entrained whereas metabolic pathways related to neuronal dysfunction and hypoxia are phase advanced. Further studies are now required to evaluate the chronic implications of such desynchronization on the development of diabetic retinopathy.

## Graphical Abstract

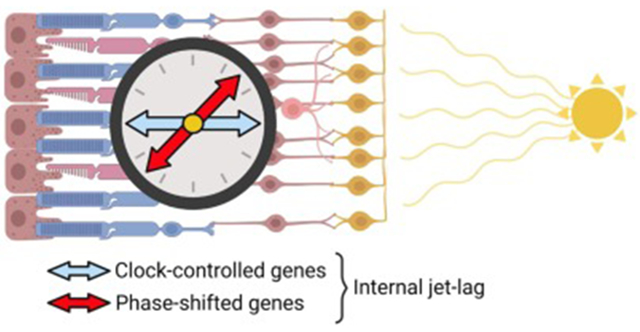

## 1. Introduction

With the inexorable increase in the incidence of diabetes, it is estimated that by 2040 around 224 million people will have some form of diabetic retinopathy (DR), with approximately 70 million suffering severe vision loss (International Diabetes Federation, 2019). Current treatments are administered only at the late stages of the disease and do not always prevent vision loss, carry side effects, and place a substantial economic burden on healthcare systems worldwide. Early diagnosis, prevention strategies, and new therapies are urgently needed. In this respect, understanding early pathobiology and how environmental inputs impact disease progression can provide the basis for designing prevention strategies by modifying these inputs.

DR pathogenesis is complex and incompletely elucidated. Hyperglycemia drives early-stage retinal gliosis, neuroinflammation, and vascular pathology, which progresses to ischemia and the sight-threatening stages typified by neuronal depletion and loss of retinal integrity due to edema and neovascularization. Current treatments target the late stages of the disease stages, despite changes in retinal biochemistry often occurring long before clinical diagnosis. Rodent models of diabetes have been instrumental in elucidating these early changes in the diabetic retina. At the early stages, clinical efforts focus on managing glucose levels and blood pressure but proportionately more provision for lifestyle or environmental changes could benefit patients. Modeling disease progression in rodent models of diabetes represent only the early stages of DR. Vascular pathology in the diabetic models, presents as increased vascular permeability and inflammation at 2 months of diabetes duration and as loss of vasculature starting around 6 months of diabetes (Veenstra et al., 2015). However, these models have also highlighted that neuronal dysfunction and gliosis is another impact of diabetes on the retina that has not been described in the clinic and could offer significant insights in the pathobiology of the disease.

One emerging system that is disrupted early after diabetes diagnosis and before the appearance of complications is the circadian clock. The circadian clock is a time-keeping mechanism that facilitates adaptation of the cellular physiology to the daily light/dark cycle. While it is well established that circadian disruption predisposes to diabetes (Hung et al., 2006; Marcheva et al., 2010; Stenvers et al., 2019; Vetter et al., 2018), disease-induced disruption of the circadian system emerges as an important factor for prognosis of disease management and outcomes. Specifically, circadian disruption in diabetes seems to impact peripheral clocks more than the master clock located in the suprachiasmatic nucleus (Busik et al., 2009; Stenvers et al., 2019) leading to a misalignment between the circadian clocks throughout the body. Additionally, a metabolic jet-lag which refers to a state of phase shift in circadian profiles of metabolic or endocrine pathways was described for people with aberrant eating behaviors (Asterholm & Scherer, 2012) but it can also occur in diabetes.

The circadian clock, a molecular mechanism, adapts multiple aspects of our physiology to the daily light cycle. It comprises a group of proteins operating in a translational transcriptional feedback loop (TTFL) that takes approximately 24 h to complete. By regulating the rhythmic transcription of genes at the cellular level, this system coordinates multiple aspects of our physiology to adapt to the daily light/dark cycles. As in all our tissues, circadian clocks operate in the retina (Baba et al., 2018; Storch et al., 2007; Tosini & Menaker, 1996) and prepare it for the metabolic and functional changes of the day-night cycle. There is clear evidence that diabetes affects retinal circadian clock rhythms and alters circadian gene expression in the retina in both type 1 and type 2 diabetic models (Busik et al., 2009; Di et al., 2019; Lahouaoui et al., 2014, 2016; Qi et al., 2020; Wang et al., 2014). Importantly, this disruption occurs before the appearance of morphological changes associated with a diagnosis of DR (Busik et al., 2009).

While these studies indicate clearly that diabetes disrupts how this molecular mechanism works, light can entrain the clock daily. At least one study indicates that light-induced circadian gene expression in the retina is also affected in diabetes (Lahouaoui et al., 2016) due to reduced c-fos and dopamine release upon light stimulation. Additional studies indicate that under physiological light/dark conditions, the daily rhythms of activity, blood pressure, and temperature (Grosbellet et al., 2016; Su et al., 2008), the rhythms of glucose and insulin sensitivity (Boden et al., 1996; Shapiro et al., 1991) circulating immune cells (Beam et al., 2021), metabolites (Beli et al., 2019; Isherwood et al., 2017) and the microbiota (Beli et al., 2019; Reitmeier et al., 2020) are impaired by diabetes.

This study was designed to map the gene expression changes in control and diabetic retinas under physiological light/dark conditions that correspond to what diabetic patients experience in their daily life. Retinas were collected from four-month-old Ins2^Akita^ mice at different time points in the day/light cycle and global mRNA sequencing performed. We confirmed that functional compartmentalization of transcriptional rush-times takes place, with a 12hr phase difference in day and night present in the retina. Interestingly, we showed that very early in diabetes and before the appearance of DR pathology, phase advances in pathways regulated by HIF1A were identified. In contrast the circadian clock remained in phase and entrained to the light/dark cycle. We suggest that this leads to an internal desynchrony between metabolic and circadian-led rhythms that could contribute to the pathogenesis of DR.

## 2. Methods and materials

### 2.1. Ethics statement

Animal studies were carried out at the institutional animal care facilities at the Indiana University School of Medicine per institutional and national guidelines for the care and use of laboratory animals (IACUC #10604 and #11167).

### 2.2. Mouse model

The C57BL/6-Ins2^Akita^/J (Ins2^Akita^) mouse model of T1D was selected in this study. Heterozygous male Ins2^Akita^ mice develop severe hyperglycemia by 4.5 weeks resulting from insulin misfolding and beta cell destruction. The phenotype is more severe in males than females (Barber et al., 2005; Yoshioka et al., 1997) and male mice were therefore used for this study. Mice were bred at Indiana University according to approved IACUC. Male Ins2^Akita^ heterozygous mice were bred with female C57Bl6 mice, and heterozygous male Ins2^Akita^ exhibited consistent hyperglycemia (>250 mg/dL), while wild type littermate males were used as controls. Mice were kept at constant temperature and humidity under a 12 h:12 h Light/Dark (12L/12D) schedule from birth, with *ad libitum* access to food and water, and when they reached four months of age were euthanized for sample collection. This age was chosen as a narrow window where mice have established diabetes but still do not develop overt DR, which occurs after six months of age (Barber et al., 2005). Sampling was performed at different times of the day/night cycle.

### 2.3. Sample collection

**Fig. 1.**
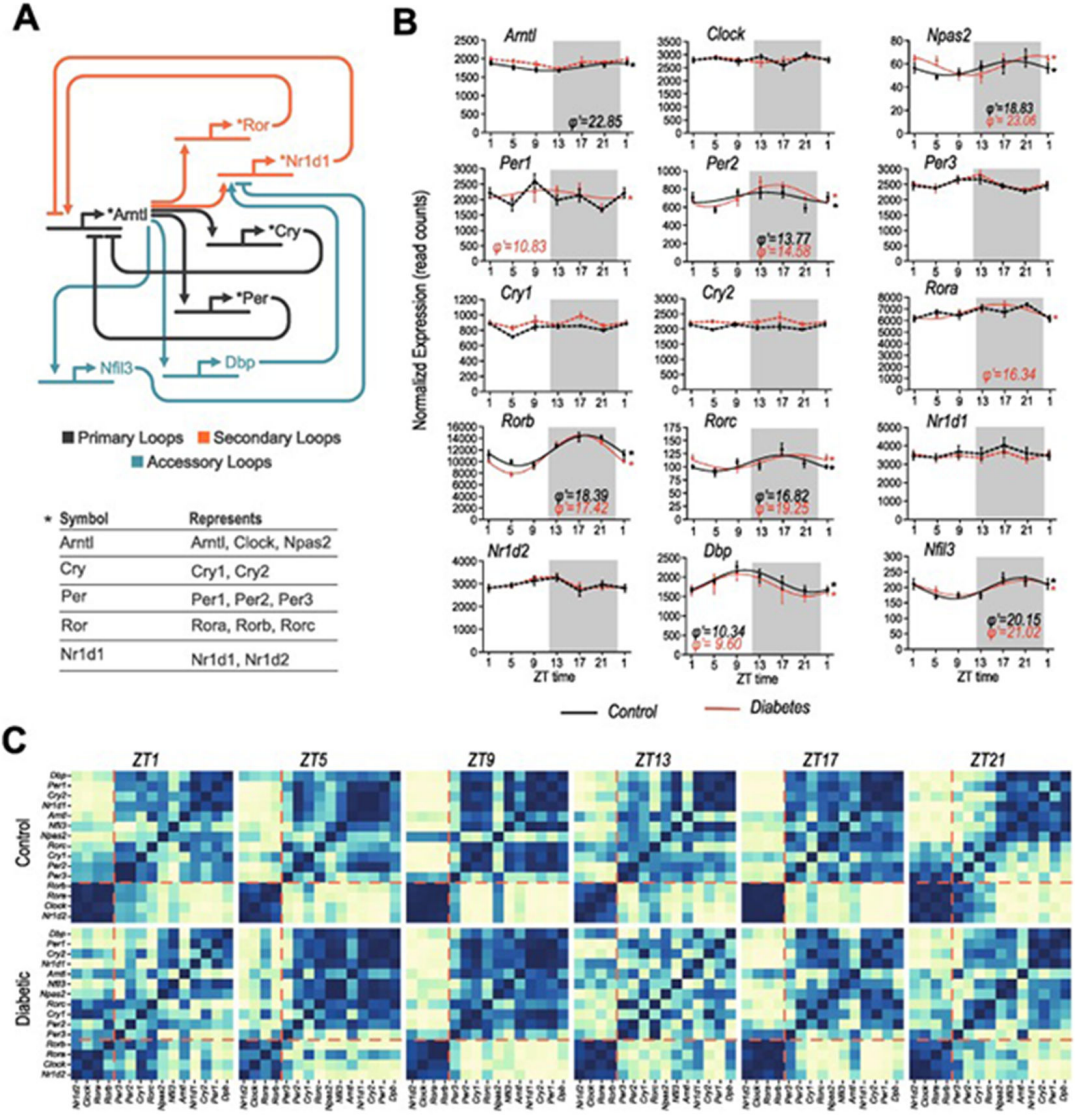
Clock Gene expression in the retina. (A) Simplified schematic of the molecular clock, showing some of the main interactions of each gene, including if they inhibit or promote gene expression. For simplicity, genes with similar interactions are represented by a single symbol in the diagram, indicated by the table at the bottom. Primary, secondary, and accessory loops are additionally indicated. (B) Expression patterns of clock genes in control (black) and diabetic (red), (mean and standard error for each time-point). Clock genes that have been identified as rhythmic by eJTK cycle package, (emp < 0.05) are indicated by an asterisk and solid lines and their respective phase in hours (φ′) beneath. Broken lines indicate no detected rhythmicity. (C) Spearman’s rank correlations between each of the 15 clock genes in both control and diabetic groups. Blue indicates strong correlations and yellow/white weakened correlations.

Mice were selected randomly at four months of age and euthanized at one of six time-points over a 24 h light/dark cycle (ZT: 1, 5, 9, 13, 17, 21 where ZT0 is indicative of lights on and ZT12 lights off), with 4–5 biological replicates for each time-point (S. Fig. 1). Terminal perfusion was conducted using cold phosphate-buffered saline (PBS) solution (without Ca^2+^ and Mg^2+^), eyes enucleated, and retinas were dissected under a microscope. For the night points (ZT12-ZT24), all processes were performed under dim red light. Following isolation, retinas were immediately frozen in liquid nitrogen and stored at −80 °C. Processing of samples took ~10 min for each sample and ~1 h for each ZT group, starting 30 min before the time-point and finishing 30 min after. Frozen samples were shipped to Queen’s University Belfast for preparation and sequencing.

### 2.4. Sample preparation and sequencing

For individual retinal samples, total RNA isolation was performed using the Qiagen RNeasy Mini Kit. RNA quality was evaluated prior to RNA sequencing with the Agilent 2100 Bioanalyzer. Complementary DNA (cDNA) libraries were constructed using the KAPA RNA HyperPrep Kit with RiboErase (HMR) amplification and quality control procedures conducted for individual cDNA libraries. Generated Libraries were quantified using Kapa Quantification, normalized, and pooled in equimolar amounts, and quality control was performed. RNA seq was conducted in the Queens’ University Belfast Genomics Core Technology Unit (https://www.qub.ac.uk/sites/core-technology-units/Genomics/) using 100-cycle Illumina NovaSeq 6000 S1 paired-end (2x50bp) sequencing, which produced an average read depth of approximately 27.5 million reads per sample.

### 2.5. Read processing, mapping, and quantification

Raw sequence reads for diabetic and healthy control retinas were initially inspected for quality using FastQC (Andrews, 2010). Raw sequence reads were mapped to the *Mus musculus* reference genome (GRCm38.p5) and annotated using Gencode (M15) with the STAR aligner program (v2.7.4a) (Dobin et al., 2013). HTSeq-count (v0.11.1) (Anders et al., 2015) was used to generate the raw counts for each sample, and the resultant read count matrix was normalized together using DESeq2 (v1.26.0) (Love et al., 2014). To increase statistical power for identifying truly cycling gene features with extremely low read counts (Hughes et al., 2017), following normalization and visual inspection of the data, genes with a normalized median read count > 4 across all samples were considered expressed and included for further analysis. The average read count was 31,758,612 reads (S. Table 1), and annotated genes expressed in control and diabetic retinas are listed in (S. Table 2).

### 2.6. Identification of cycling genes

Genes displaying an approximately 24-hour rhythm were identified using the nonparametric rhythmicity detection algorithm empirical JTK_CYCLE with asymmetry search (empJTK) (Hutchison et al., 2015). Briefly, empJTK is an advancement on the well-accepted JTK_CYCLE rhythmicity detection method, which allows the identification of transcripts displaying asymmetric waveforms and is considered one of the most robust methods for identifying periodic patterns in gene expression data (Laloum & Robinson-Rechavi, 2020). Normalized diabetic and control read count matrices were formatted as a single cycle with 4–5 biological replicates for each timepoint, and both phase and asymmetry searches were set to 4-hour intervals ranging from ZT0 to ZT20. Significantly cycling genes were defined as those with an empJTK empirically calculated p-value (empP) < 0.05 and an empJTK predicted min/max fold-change of >1.25. empJTK outputs for all datasets analyzed are found in S. Table 3. The Metascape online tool (Zhou et al., 2019) was additionally used to compare multiple gene lists.

### 2.7. Acrophase prediction

Since empJTK is only effective at predicting acrophases no more densely than the resolution of the data, harmonic regression was subsequently used to accurately predict the acrophases of significantly cycling genes using the Harmonic Regression R package (v.1.0) with a fixed period of 24 hr and normalization set to false. Radian acrophases (*φ*) were then converted to hours (*φ*′) using the equation *φ*′ = (−*P*/*2π*)*φ*, where *P* is the pre-set period of rhythmicity (24hr) (Refinetti et al., 2007). Acrophases for all rhythmic genes are listed in S. Table 3.

### 2.8. Functional annotation and phase set enrichment analysis

To analyze the functions of cycling genes while considering the acrophase of the transcript, a DO and KEGG phase set enrichment analysis (PSEA) (Zhang et al., 2016) was carried out using gene sets from the Molecular Signatures Database (MSigDB) (Subramanian et al., 2005). Any GO or KEGG term with a BH-corrected p-value < 0.01 was significantly enriched. Data is shown in S. Table 4. Harmonic regression predicted acrophases were rounded to the nearest hour. Testing was conducted on a uniform background using the Kuiper test. If sets contained less than ten transcripts, they were excluded from the analysis.

### 2.9. Differential rhythmicity detection

Differential gene expression at each time points and over all time points was performed using DESeq2 (Love et al., 2014). Differentially expressed transcripts were defined as those with a Benjamini-Hochberg adjusted p-value < 0.05. Data are shown in S. Table 5. To test for rhythmicity changes in amplitude and phase of genes that had been identified as significantly cycling in either diabetic or control retinas, the DODR R package (v0.99.2) was used, (Thaben & Westermark, 2016). Normalized read counts were used with the robust DODR method with a fixed period of 24 hrs. The resultant DODR p-values were Benjamini-Hochberg (BH) adjusted for multiple testing, and an adjusted p-value < 0.05 was used to define differential rhythmicity. Differentially rhythmic transcripts were then further subdivided into those displaying a phase shift when the difference in acrophase between diabetic and control > 1 h and those displaying a substantially altered amplitude with absolute log_2_ amplitude fold-change > 0.32. Genes identified as differentially rhythmic are listed in S. Table 6.

### 2.10. Qiagen ingenuity pathway analysis (IPA)

The list of differential rhythmic genes was used for IPA Core Analysis and canonical pathway analysis. The graphical summary of the results was used to understand how the pathways are inter-related to one another. Lists of genes that showed significant phase shifts or amplitude changes were used for upstream regulator analysis to understand the possible upstream factors that drive either of those changes.

### 2.11. Data availability

The RNA-seq data reported in this article has been deposited in NCBI’s Gene Expression Omnibus (GEO) and is accessible through GEO Series accession number GSE233440.

## 3. Results

### 3.1. Daily expression of clock genes in the diabetic retina

**Fig. 2.**
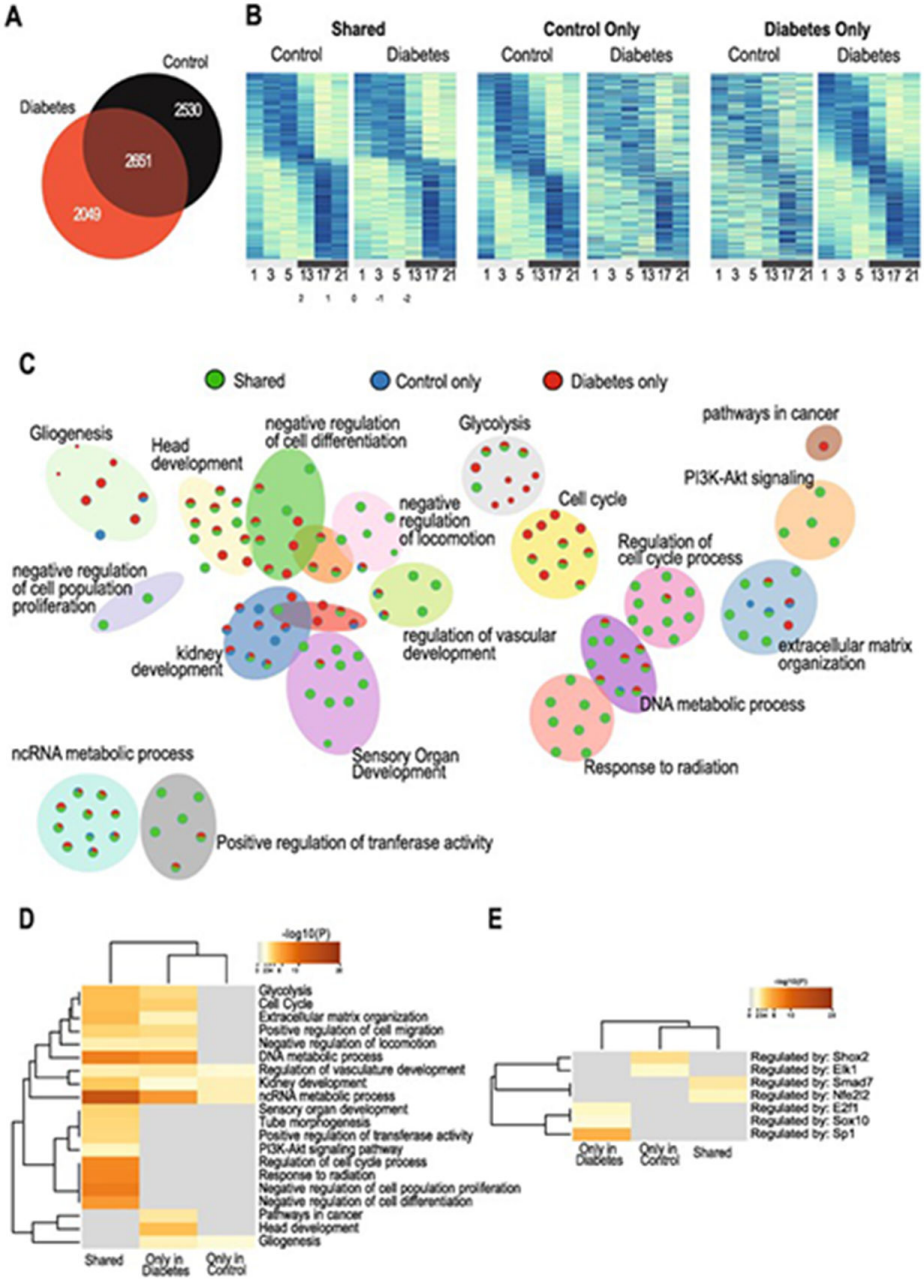
Disorganization of rhythmic transcripts in diabetes. (A) Venn diagram showing the overlap between transcripts identified as rhythmic in the control and diabetic retinas. (B) Heatmaps indicate loss, gain or retention of rhythmicity in control and diabetic retinas. Transcripts expression were min/max normalized and sorted by harmonic regression predicted acrophase. Each row represents a gene identified as cycling in each control or diabetic or both. Each column represents a single time-point, where biological replicates expression was averaged. Blue to yellow hue represents gene expression from max to min. (C) Metascape network pathway analysis of transcripts that were identified as rhythmic. Nodes are coded with colors depending on if they represent rhythmic genes found only in control (blue), only in diabetic (red), or shared in both control and diabetic retinas (green). (D) Gene set enrichment analysis revealing the pathways identified to be rhythmic only in diabetes, only in control or shared. (E) Predicted regulators for the rhythmic genes in each category.

A list of 15 known clock genes from the primary (*Arntl, Clock, Npas2, Per1, Per2, Per3, Cry1, Cry2*), secondary (*Nrd1d1, Nr1d2, Rora, Rorb, Rorc*), and accessory TTFL loops (*Nfil3, Dbp*) (Fig. 1A) were selected to investigate the effect of diabetes on their rhythmic expression. Seven clock genes were identified as rhythmic in the retinas from the control group (*Arntl, Npas2, Per2, Rorb, Rorc, Dbp, Nfil3*) and eight in the retinas of the diabetic group (*Npas2, Per1, Per2, Rora, Rorb, Rorc, Dbp, Nfil3*) (Fig. 1B). Overall, these data showed limited effects of diabetes on clock gene expression in the retina. Yet, *Arntl1* was rhythmic only in controls, while *Per1,* and *Rora* only in diabetic retinas. While *Per2* and *Rorb* had slightly increased amplitude, notably, the phase of most clock genes in the retina did not change, except for *Rorb* and *Npas.* Since the retinal clock is entrained by the light/dark cycle, differences in the phase under these conditions were not expected. To confirm this, we then analyzed several known clock-controlled genes for the retina (Storch et al., 2007; Vancura et al., 2021) (*Adcy1, Drd4, Nr2e3, Cys1, Plekbh1, Usp2*) (S. Fig. 2) that also showed amplitude changes but no phase changes, indicating that the retinal clock was entrained to the 12L:12D cycle at this stage of diabetes.

Due to the nature of transcriptional/translational feedback loops, clock gene expressions are either directly or indirectly connected and a level of correlation would be expected between genes. For example, when *Arntl* expression is increased, the expression of its repressors, *Per, Cry, Reverba,* should be low. Spearman correlations were calculated between each of the 15 molecular clock genes for all of the timepoints to reveal whether there is disruption in the global expression patterns of all clock genes. While in control retinas, clock gene correlations could be separated into two main groupings, correlations were weakened in the diabetic retinas, most prominently at ZT1 and ZT13 (Fig. 1c). On closer inspection, correlation disruption can also be identified at other time-points, as the *Npas2* correlations at ZT9 being strengthened for some genes and weakened for others. Overall, these data indicate a certain level of disruption in the clock machinery in the diabetic retina that might be related to its daily entrainment by the light as these are more evident at dawn and dusk.

### 3.2. Global daily rhythmic transcriptome in the diabetic retina

Next, we compared the global rhythmic output in control and diabetic mice. 4,700 transcripts in control mice and 5,177 transcripts in diabetic mice were identified as rhythmic using empJTK (S. Table 3). When compared to the total number of transcripts identified as expressed (>4 read counts) in the control (Boden et al., 1996) and diabetic retinas (Boden et al., 1996), 25 % of expressed transcripts in control and 27 % in diabetic retinas displayed rhythmic expression.

In contrast to the subtle effects identified for the clock genes, the level of disruption by diabetes on the global rhythmic transcriptome was significantly greater. Of all rhythmic genes, only 36.7 % (Veenstra et al., 2015) transcripts were shared between control and diabetic retinas, while 28.4 % (Veenstra et al., 2015) gained *de novo* rhythmicity and 35 % (Veenstra et al., 2015) lost diurnal rhythmicity (Fig. 2A). A distinct “gain” or “loss” of rhythmicity can be viewed in the heatmap (Fig. 2B). Pathway enrichment analysis of the genes found rhythmic only in control or only in diabetes or shared, revealed three major networks being impacted, one related to development and differentiation, another to cell cycle, damage, and metabolism, and a third on PI3K-AKT signaling (Fig. 2B). The pathway heatmap (Fig. 2C) revealed that most of the pathways were shared, but a lot of these were found to lose rhythmicity in diabetes, such as organ development, PI3-AKT signaling pathway, regulation of cell cycle, response to radiation, cell population proliferation and differentiation, with pathways in cancer and head development only found to be rhythmic in diabetes (Fig. 2D). E2f1, Sox10 and SP1 were found to likely regulate the expression of genes found only in diabetes (Fig. 2E).

**Fig. 3.**
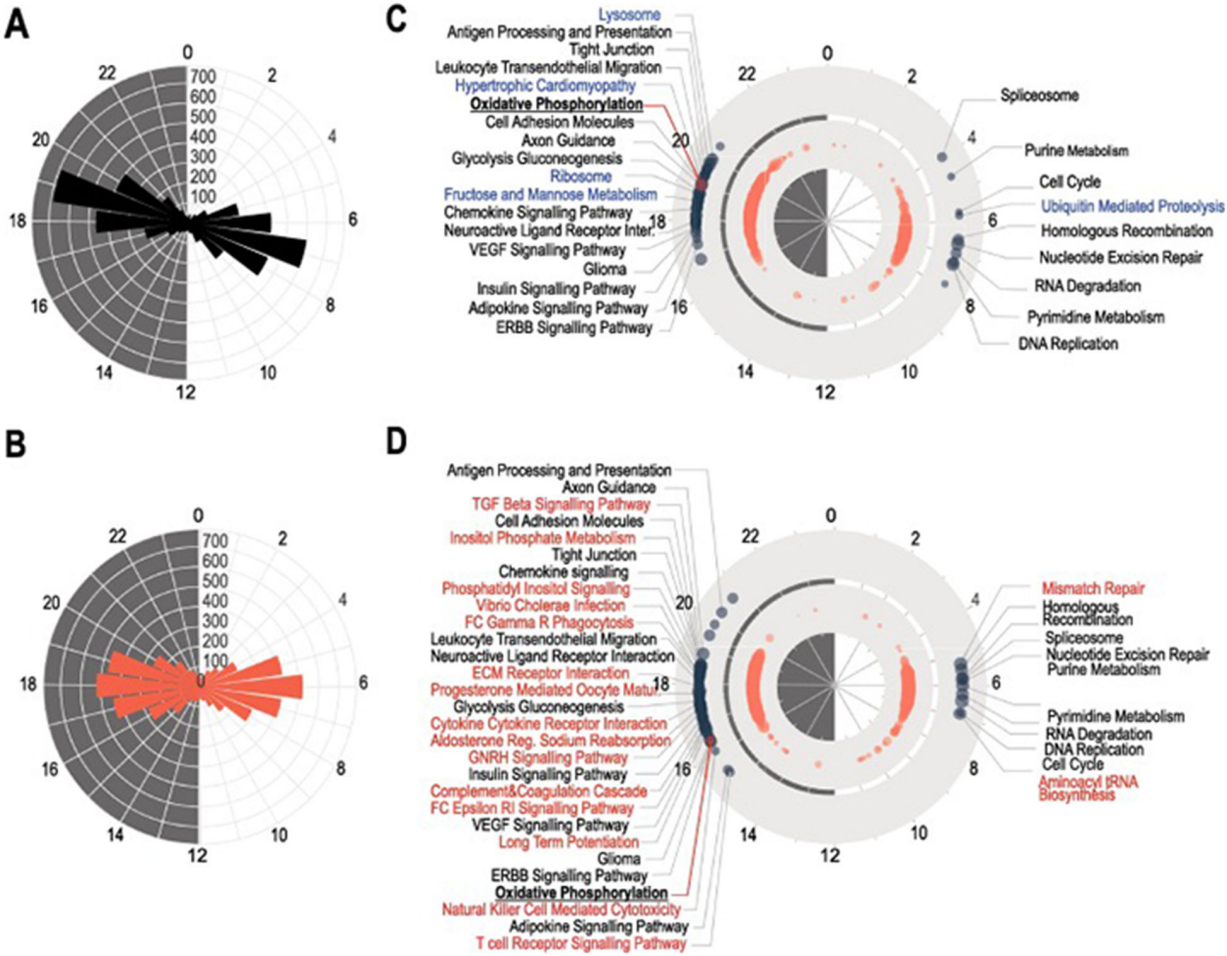
Phase differences in rhythmic transcripts. (A–B) Radial plots of the distribution of the peak phase of rhythmic transcripts in control (A), and in diabetic (B) retinas. (C–D) Phase distribution over the 24 h cycle of representative KEGG pathways in control (C) and diabetic (D) retinas. Phases were calculated using Set Enrichment Analysis (PSEA) for rhythmic transcripts. KEGG pathways which were uniquely identified as rhythmic in control are highlighted in blue and in diabetes in red.

Plotting the time of the acrophases, determined by harmonic regression, revealed two clear peaks in transcription in both control and diabetic retinas, one during the day and one during the night, separated by an approximately 12-hour difference (Fig. 3A-B). In control retinas, most transcripts peaked between ZT6 and ZT8, and ZT18 and ZT20 (Fig. 3A). However, in the diabetic retinas although the 12-hour expression axis was retained, the transcripts were slightly phase advanced to between ZT5 and ZT7 and ZT17 and ZT19 (Fig. 3B). In addition, the two peaks in the diabetic group were more widely distributed, with neither as prominent as those in control retinas. These patterns were true for both coding and non-coding transcripts.

We then conducted a functional analysis whilst considering the acrophases of rhythmic transcripts (Zhang et al., 2016). This identified that the peak of expression during the day and during the night have separate roles in the retina (Fig. 3 C-D). Genes peaking at the mid-day were enriched in pathways related to cell cycle, DNA replication and RNA degradation, while those that peaking at mid-night were enriched for metabolism, inflammation and growth factor signaling pathways, indicating a clear functional compartmentalization of the retinal transcriptome between day and night. Interestingly, there were fewer enriched KEGG pathways during the day when compared to the night. Most importantly, we identified unique KEGG pathways which were enriched in either the control or the diabetic retina. During the day, control retinas (Fig. 3C) showed enrichment of the ubiquitin-mediated proteolysis pathway genes which was lost in diabetes. In contrast, diabetic retinas had enriched pathways related to mismatched repair and tRNA biosynthesis pathways not found to be rhythmic in control retinas (Fig. 3D). During the night, more pathways were altered by diabetes, especially those related to inflammation, hormonal and cytokine signaling, while a few were absent, such as ribosome, fructose and mannose metabolism, lysosome and pathways related to cardiomyopathy (Fig 3D) and S. Table 4. The genes in some pathways exhibited an even earlier peak phase in diabetes than the total cycling transcripts. Like the earlier peak phase of the total cycling transcripts, KEGG pathways also exhibited an earlier peak in diabetic retina compared to control, but some more than others. Oxidative phosphorylation, for example, was advanced by almost 4 h in diabetes compared to control.

### 3.3. Amplitude and phase changes in the rhythmic transcriptome in the diabetic retina

We first aimed to identify whether diabetes resulted in differential expression at individual timepoints (ZT1, −5, −9, −13, −17, −21) or among all samples in control and diabetic groups independent of the time, but we only identified few genes been significant different with these approaches (Sup. Table 5). The very low numbers of differentially expressed transcripts perhaps indicates there were no differences in the relative expression of each transcript, although they may display changes in rhythmicity patterns.

**Fig. 4.**
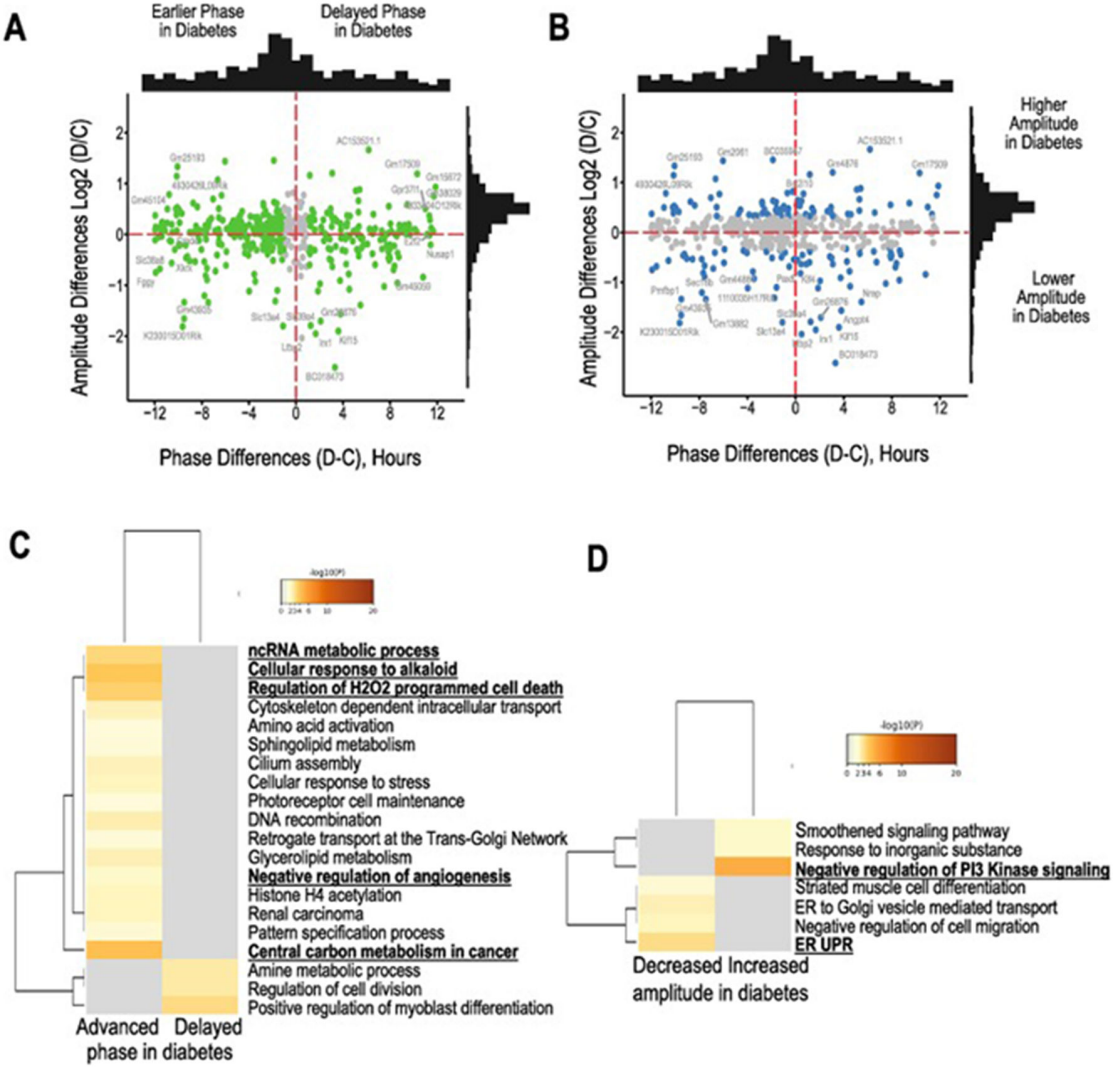
Phase and amplitude changes of differentially rhythmic transcripts between controls and diabetic retinas. DORD analysis was performed to identify differential rhythmic transcripts based on amplitude and acrophase. (A) All transcripts that showed a statistically significant difference in phase are highlighted in green with the distribution of the number shown on the top. (B) All transcripts with differences in amplitude are shown in blue. A distribution of the number of genes shown on the right. (C) Differentially rhythmic genes were further subdivided into those that displayed advanced or delayed phase and Metascape analysis was performed for pathway and enrichment analysis; (D) Metascape analysis for genes that had increased or decreased amplitude in diabetes.

To further examine the differential rhythmic expression of genes between control and diabetic retinas, we used the differential rhythmicity analysis, DODR (Thaben & Westermark, 2016), and identified a total of 478 genes as differentially rhythmic (S. Table 6). We split these into 2 main groups: transcripts that displayed an altered amplitude (Fig. 4A), and transcripts that displayed a phase shift > 1-hour (Fig. 4B). A higher number of genes identified as phase-shifted (405transcripts) compared to genes with altered amplitude (154transcripts). This was also confirmed using cosinor analysis, where indeed, most of the genes showed a phase-shift in their expression rather than an amplitude change (data not shown). Most shifted genes were phase-advanced in the diabetic retinas.

Enrichment analysis of the differentially rhythmic genes (Fig. 4C) revealed that central carbon metabolism, ncRNA-regulated metabolic processes, cellular response to alkaloids and oxidative induced cell death were the most enriched pathways, while circadian control was not identified to be differentially rhythmic in the analyzed retinas. Sphingolipid metabolism and histone H4 acetylation were also among the pathways that were enriched in genes with advanced phase in diabetes. Genes that showed delayed phase were enriched in cell division and differentiation pathways. In contrast, pathways that exhibited increased amplitude with diabetes (Fig 4D) were enriched in the regulation of PI3K signaling, and those that had reduced amplitude, in endoplasmic reticulum stress and the unfolded protein response (UPR).

**Fig. 5.**
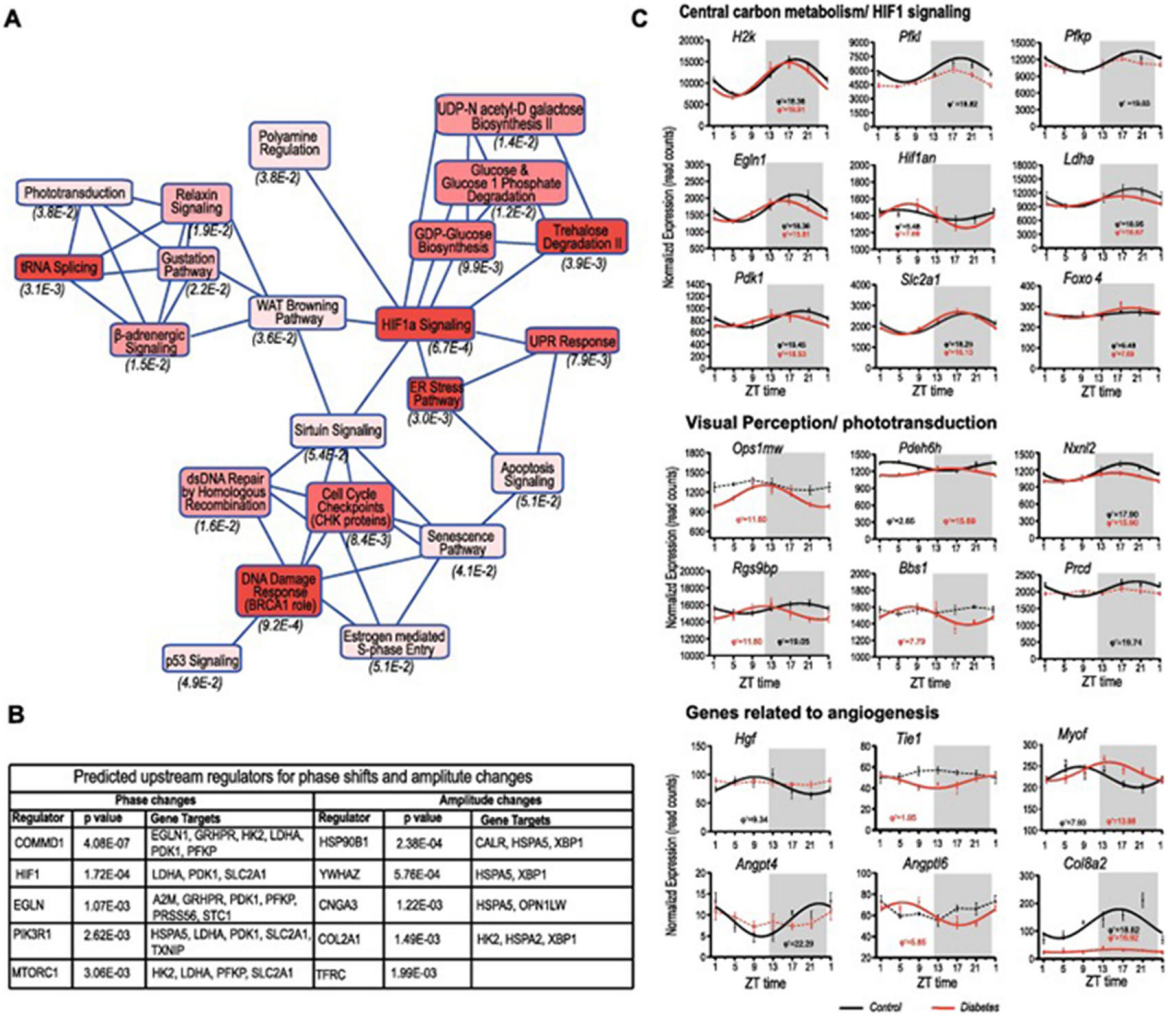
Pathway analysis of differential rhythmic genes. (A) Graphic summary of canonical pathway analysis with IPA Core Analysis illustrating how enriched canonical pathways are related to one another. (B) Upstream Regulator Analysis indicating transcriptional regulators likely to be related to phase and amplitude changes. (C) Expression patterns of genes that showed different rhythmicity in diabetic retinas compared to control, involved in central carbon metabolism and HIF1 signaling, visual perception/phototransduction and pathogenesis of DR. Genes identified as rhythmic by eJTK cycle package, (emp < 0.05) are indicated by an asterisk and solid lines and their respective phase in hours (φ′) beneath. Broken lines indicate no detected rhythmicity.

Canonical pathway analysis of all the genes that were identified to be differentially rhythmic in control and diabetes included phototransduction, glucose metabolism, HIF1a signaling, ER stress, DNA damage and cell cycle checkpoints to be affected (Fig. 5A). Upstream regulator analysis suggested COMMD1, HIF1A, EGLN, PIK3R1 and MTORC1 as major upstream regulators for the phase shifts, while HSP90B1, YWHAZ, CNGA3, COL2A1 and TFRC (Fig. 5B) were identified as the major upstream regulators for the amplitude changes observed in the diabetic retina.

We then explored some of the pathways that were identified to be driving the phase shifts. Among the genes that were differentially regulated in the central carbon metabolism and Hif1a signaling, *Hk2, Pfkl, Pfkp, Egln1, Ldha, Pdk1, Glut1* were advanced by several hours as shown in Fig. 5C. However, genes related to photoreceptors were significantly phase shifted, with *Pdhe6* and *Rgs9bp* exhibiting around 10-hour phase-shifts. This altered rhythmicity involved some genes related to angiogenesis, including *hgf* (Boulton, 1999) *Tie1* (La Porta et al., 2018), *Myof* (Yoshida et al., 2010) *angpt4, angptl6* (Whitehead et al.,2019); and *Col8a2* (Hwang et al., 2020), involved in pathways that may be associated with DR. Overall, these data indicate that a possible metabolic dysfunction involving metabolism and visual pathways around the daily cycle drives the phase shifts observed in the diabetic retina.

## 4. Discussion

Emerging evidence for circadian disruption in the diabetic retina (Busik et al., 2009; Di et al., 2019; Lahouaoui, Coutanson, Cooper, Bennis, & Dkhissi-Benyahya, 2016; Wang et al., 2014) prompted us to perform a detailed transcriptomics analysis in the retina of diabetic mice under real-life conditions (light/dark) rhythmicity. We used the Ins2^Akita^ mice, a hyperglycemic model of type 1 diabetes that exhibits vascular, neural, and glial abnormalities consistent with clinical observations of early diabetic retinopathy (Barber et al., 2005). Retinas were taken at early stage (4 months of age, 2 months of diabetes) when retinal complications such as increased vascular permeability and the first neuronal abnormalities are observed, but before abnormal vascular pathology (Barber et al., 2005). Deep mRNA sequencing was performed for retinas collected around the daily light/dark cycle to examine the effects of early diabetes in the daily rhythmic output of the retinal transcriptome. Our results confirmed that a 12-hour axis in transcriptional compartmentalization between day and night is present in the retina, as reported by others (Wang et al., 2021) and for other organs (Mure et al., 2018). This axis remained in diabetes but was phase-shifted by 1–3 hrs, or up to 10 hrs or more for some individual genes. An advancement of phase rather than delay or change in amplitude was mostly observed in the differential rhythmic genes. Interestingly, these phase shifts seem to be mainly driven by the phase advance observed in pathways related to metabolic dysfunction and visual pathways, involving HIF1a and mTOR1 signaling pathways, rather than alterations in the phase of the circadian retinal clock, suggesting that in early diabetes an internal desynchronization occurs in the retina.

While it is established that diabetes distorts clock gene expression in the retina (Busik et al., 2009; Lahouaoui, Coutanson, Cooper, Bennis, & Dkhissi-Benyahya, 2016; Wang et al., 2014), the current study shows that under light/dark conditions only subtle changes in the amplitude and the phase of expression of clock genes were detected. Clock genes remained in phase, except for *Npas2,* but *Arntl1* lost while *Per1* and *Rora* gained rhythmicity. A degree of discoordination of clock gene relationships was observed in diabetes, especially at dawn and dusk, indicating subtle effects of diabetes in the synchronization of the clock machinery to light. While our data suggest that clock gene expression remained in phase, we do not exclude the possibility that specific cell clocks are influenced by diabetes, nor that the subtle changes are due to loss of specific cell populations within the diabetic retina or alterations of clock regulation within the retina. The transcriptome in bulk mRNA sequencing represents mostly rod photoreceptors (65 %), cones by 5 % and neurons of the inner retina for 25 % with only the rest 5 % from non-neuronal cells (Wang et al., 2021). Therefore, this approach can mask changes in clock gene expression in specific cell-types that have proportionately low representation within the retina, such as endothelial cells. Single cell transcriptomics analysis would be more appropriate to define the effects of diabetes on the individual cellular clocks in the retina, especially since this tissue is heterogeneous with different phases of the clock at different layers (Dkhissi-Benyahya et al., 2013; Jaeger et al., 2015). Although this was beyond the scope of this study, identifying how diabetes impacts the interconnections of the cellular clocks within the retina is a very interesting area that requires further investigation. Our study so far shows that in this early time point and under entrainment conditions, clock gene expression remained in phase, even if they exhibited subtle changes in amplitude which could be driven by cell death, or impact on the clock regulation itself.

Few studies exist that have analyzed the diurnal or circadian transcriptome in the murine (Storch et al., 2007; Wang et al., 2021), primate (Mure et al., 2018), drosophila (Damulewicz et al., 2018) and zebrafish (Ramasamy et al., 2019) retina, none of which were done in the context of diabetes. Our study confirms recent results from Wang et al. (2021) that performed mRNA sequencing in healthy murine retinas. In that study it was also shown that metabolic genes with oxygen sensing, carbon metabolism and HIF1A signaling were at the core of the night peaking genes, while day peaking genes were related to ribosome biogenesis and non-coding RNA processing as we also observed. These same night peaking pathways are at the core of the phase advancements we observed in the diabetic retina. Moreover, the phase advance observed in the diabetic retinas agrees with a previous study by Jiao et al. (2020) performed in extra-orbital lacrimal glands. In addition, the latter study as well as ours, observed little overlap between the rhythmic genes in control and diabetes. Similarly, Vancura et al. (2021) that performed a microarray in the retina of T2D mice (db/db) comparing two time points (one at day and one at night) identified a small overlap of the common genes that change from day to night, indicating a major reorganization of the daily rhythmic transcriptome by diabetes. We further identified that there were more genes gaining rhythmicity in diabetes compared to control, similarly with Jiao et al. data (Jiao et al., 2020). Genes that gained “de novo” rhythmicity due to diabetes belonged to pathways of responses to growth factors, transmembrane receptor protein kinase signaling, negative regulation of cell proliferation and cellular component organization, positive regulation of cell death and head development. The gaining of rhythmicity for these genes may not be driven by the circadian clock itself but by the disease, but it does reveal a topological preference in the temporal timeline of a day, bringing into focus the application of chronotherapies for the management of DR since most of the genes peaking during the night and therefore gaining rhythmicity are active targets for the management of DR.

Our analysis revealed that the retina exhibits a 12 hr transcriptional compartmentalization, which is induced by light and fine-tuned by the circadian clock. In agreement with the Wang et al. (2021) study, day peaking genes favored DNA repair, RNA splicing and ribosomal protein synthesis, whereas night peaking genes are related to metabolic processes and growth factor signaling and the major therapeutic targets for DR. However, our study revealed that although the 12 hr transcriptional axis was retained in the diabetic retina, it was phase -advanced by approximately 1–3 hr with a wider distribution. Interestingly, we found more alterations in the phase than in the amplitude of the rhythms, something that was also observed in other ocular tissues in diabetes as well (Jiao et al., 2020). This may increase the susceptibility of the retina to certain challenges, such as the response to light damage. Moreover, even when small phase misalignments with the daily cycle are imposed, the damage to the tissue may accumulate when these conditions become chronic. For example, a chronic small phase misalignment of the internal clock with the external light/dark cycle can have severe impact on the metabolic and cardiovascular health of mice (West et al., 2017). Although we suggest that this may not be driven by the circadian clock under light/dark conditions, previous studies in diabetic models indicate that the retina circadian clock in diabetes is disrupted (Busik et al., 2009; Lahouaoui et al., 2016; Wang et al., 2014). Of interest is a study in a type 2 diabetic Per2::luciferase mouse which suggests that the circadian period of many peripheral tissues, such as the liver and white adipose tissue, is significantly reduced and therefore circadian rhythmicity is phase - advanced (Hou et al., 2019).

Our findings led us to propose a possible mechanism that could facilitate the damage occurring in the diabetic retina. Diabetes results in an internal desynchronization in the retina driven by a phase advancement of pathways related to hypoxia and metabolism, while the circadian clock rhythms remain entrained to the light/dark cycle, driving clock-controlled responses in phase with the environment. The implications of these phase shifts are not yet known, but we hypothesize that the chronic internal desynchronization, observed in the early stages of diabetes, contributes to the pathology of DR by increasing oxidative stress and neuronal damage early on. The implication of such desynchronization is that having a functional and entrained retinal clock in diabetes might have a pathogenic role for the progression of DR. Indeed, a pathological role of Bmal1 in the progression of DR was recently proposed by Vancura et al. (2021), who showed that Bmal1 deletion spared the retina from the effects of diabetes. Moreover, recent work from Jidigam et al. (2022) showed that the neuronal clock in the context of pathological neovascularization is detrimental and its deletion led to protection from aberrant angiogenesis. By extension from our hypothesis, when this internal jet lag is corrected, for example, by deletion of the retinal circadian clock with retina specific knockout of Bmal1 or with correction of the metabolic phase shifts, then the progression of DR could be slowed. Future studies will be required to confirm this, but disease mediated disruption of circadian rhythms seems likely to emerge as an important risk factor in disease progression (Jidigam et al., 2022; Vancura et al., 2021).

## Supplementary Material

Supp Data 1

Supp Data 2

## Data Availability

The RNA-seq data reported in this article has been deposited in NCBI’s Gene Expression Omnibus (GEO) and is accessible through GEO Series accession number GSE233440.
